# HIV Testing, Linkage to HIV Medical Care, and Interviews for Partner Services Among Youths — 61 Health Department Jurisdictions, United States, Puerto Rico, and the U.S. Virgin Islands, 2015

**DOI:** 10.15585/mmwr.mm6624a2

**Published:** 2017-06-23

**Authors:** Renee Stein, Wei Song, Mariette Marano, Heta Patel, Shubha Rao, Elana Morris

**Affiliations:** 1 Division of HIV/AIDS Prevention, National Center for HIV/AIDS, Viral Hepatitis, STD, and TB Prevention, CDC.

Identifying persons living with human immunodeficiency virus (HIV) who are unaware of their infection, linking them to HIV medical care, and reducing health disparities are important national goals (*1*). Of the 8,841 teens and young adults aged 13–24 years (collectively referred to as youths in this report) who received a diagnosis of HIV in 2014, 70% were young men who have sex with men (MSM) (*2*). In the same year, an estimated 52% of young MSM living with HIV were unaware of their infection compared with 15% among all persons living with HIV (*3*). An average of 22% of high school students who have had sexual intercourse and 33% of young adults (persons aged 18–24 years) reported ever receiving an HIV test (*4*). CDC recommends screening all persons aged 13–64 years, with annual rescreening for persons at high risk for HIV infection (*5*). Analysis of CDC-funded program data for youths submitted by 61 health departments in 2015 revealed that young MSM, who accounted for 83% of new diagnoses among all youths in non–health care facilities, received 28% of HIV tests.* The 2020 national goal is to link at least 85% of HIV-positive persons to HIV medical care within 30 days of diagnosis. In this analysis, 66% of youths who received positive test results for HIV infection were linked to care within 90 days of diagnosis. Increasing the number of youths at risk for HIV infection who are tested for HIV on a regular basis and ensuring that youths who receive positive test results for HIV are rapidly linked to and retained in appropriate medical care, including early initiation of antiretroviral therapy, are essential steps for reducing HIV infection in this vulnerable population.

In 2015, CDC funded 61 state and local health departments and 123 community-based organizations (CBOs)^†^ to provide HIV testing and related services in the United States, Puerto Rico, and the U.S. Virgin Islands. Health departments submitted deidentified program data about services provided by both health departments and CBOs, through a secure, online, CDC-supported system. Data from 2015 analyzed for this report include CDC-funded HIV tests,^§^ new positive diagnoses, linkage of persons with newly or previously identified HIV to medical care within 90 days of diagnosis,^¶^ and interviews for partner services** among youths. Data were stratified by the following demographic characteristics: age group, race/ethnicity, gender, test setting, census region, and health department jurisdiction’s HIV prevalence.^††^ Tests performed in non–health care facilities were stratified by target population.^§§^ Multivariate log-binomial regression was used to assess the association between demographic characteristics and newly diagnosed HIV infections, linkage to HIV medical care, and interviews for partner services. To analyze linkage to HIV medical care among previously diagnosed HIV-positive youths, modified Poisson regression analysis was used, and the association between the target population and newly diagnosed HIV infections in non–health care facilities was assessed using univariate log-binomial regression.

Among 3,026,074 CDC-funded tests provided in 2015, a total of 838,342 (28%) were provided to youths. Among youths, the highest percentages of tests were provided to persons who were aged 20–24 years (74%), female (55%), and black (50%) and were provided in health care facilities (76%), in the South (58%), and in medium and high prevalence jurisdictions (97%). The highest percentages of tests performed in non–health care facilities were provided to young heterosexual females (36%), young heterosexual males (30%), and young MSM (28%) (Table 1). Findings indicated that compared with youths aged 20–24 years, those aged 13–19 years were less likely to have newly diagnosed infection (adjusted prevalence ratio [aPR] = 0.51). White youths (0.22%; aPR = 0.45), Hispanic/Latino youths (0.39%; aPR = 0.70), and youths of other racial/ethnic groups^¶¶^ (0.32%; aPR = 0.50) were less likely to have newly diagnosed infection than were black youths (0.44%). Tests in health care facilities were less likely to yield new diagnoses than tests performed in non–health care facilities (aPR = 0.60) (Table 1). Compared with tests performed in the South, tests performed in the Northeast were less likely to yield new diagnoses (aPR = 0.73), whereas tests performed in the West were more likely to yield new diagnoses (aPR = 1.20). Tests performed in medium and medium-low/low prevalence jurisdictions were less likely to yield new diagnoses (aPR = 0.75 and aPR = 0.62, respectively) than tests performed in high prevalence jurisdictions (Table 1). Findings in non–health care facilities indicated that compared with young MSM, transgender youths, youths who inject drugs, young heterosexual males, and young heterosexual females were all less likely to receive a new diagnosis of HIV infection (aPRs = 0.64, 0.19, 0.08, and 0.05, respectively) (Table 1).

**TABLE 1:** HIV tests and newly diagnosed HIV infections among youths,* by selected characteristics — United States, Puerto Rico, and the U.S. Virgin Islands, 2015

Characteristic	No. all HIV tests^†^	HIV tests^†^ among youths		No. all newly diagnosed HIV infections^§^	Newly diagnosed HIV infections^§^ among youths			
		No. (%)	% of subgroup		No. (%)	% of subgroup	% Positive	aPR (95% CI)
**Age group (yrs)**								
13–19	221,338	—	26.4	415	—	14.0	0.19	0.51 (0.45–0.56)^¶^
20–24	617,004	—	73.6	2,558	—	86.0	0.41	Reference
Gender**								
Male	1,535,214	374,757 (24.4)	45.0	10,531	2,661 (25.3)	89.6	0.71	Reference
Female	1,457,341	453,198 (31.1)	54.5	1,801	256 (14.2)	8.6	0.06	0.09 (0.08–0.10)^¶^
Transgender	13,097	4,299 (32.8)	0.5	187	52 (27.8)	1.8	1.21	1.66 (1.26–2.18)^¶^
Race/Ethnicity**								
White	785,623	201,135 (25.6)	25.6	2,657	440 (16.6)	15.1	0.22	0.45 (0.41–0.50)^¶^
Black or African American	1,304,956	396,327 (30.4)	50.4	5,843	1,727 (29.6)	59.3	0.44	Reference
Hispanic or Latino	647,773	159,865 (24.7)	20.3	3,253	626 (19.2)	21.5	0.39	0.70 (0.63–0.77)^¶^
Other^††^	87,176	21,081 (24.2)	2.7	335	67 (20.0)	2.3	0.32	0.50 (0.39–0.64)^¶^
Multiple races	21,015	8,510 (40.5)	1.1	124	51 (41.1)	1.8	0.60	0.99 (0.75–1.31)
Test setting**								
Health care facility	2,313,742	633,088 (27.4)	75.8	7,623	1,665 (21.8)	56.3	0.26	0.60 (0.56–0.65)^¶^
Non–health care facility	703,890	202,181 (28.7)	24.2	4,860	1292 (26.6)	43.7	0.64	Reference
**U.S. Census region**								
Northeast	480,665	125,699 (26.2)	15.0	1,888	373 (19.7)	12.6	0.30	0.73 (0.65–82)^¶^
Midwest	419,516	126,792 (30.2)	15.1	1,549	439 (28.3)	14.8	0.35	1.07 (0.96–1.20)
South	1,689,548	488,561 (29.0)	58.3	6,296	1687 (26.8)	56.7	0.35	Reference
West	391,353	84,675 (21.7)	10.1	2,564	431 (16.8)	14.5	0.51	1.20 (1.07–1.35)^§§^
U.S. dependent areas	44,992	12,615 (28.0)	1.5	250	43 (17.2)	1.5	0.34	1.31 (0.95–1.80)
**HIV prevalence**								
High	1,784,092	460,162 (25.8)	54.9	8,421	1,923 (22.8)	64.7	0.42	Reference
Medium	1,150,815	350,075 (30.4)	41.8	3,830	981 (25.6)	33.0	0.28	0.75 (0.69–0.82)^¶^
Medium-low/Low	91,167	28105 (30.8)	3.4	296	69 (23.3)	2.3	0.25	0.62 (0.49–0.80)^¶^
**Overall total**	**3,026,074**	**838,342 (27.7)**	**100.0**	**12,547**	**2,973 (23.7)**	**100.0**	**0.35**	**—**
**Target population (non–health care facilities only)^¶¶^**								
Men who have sex with men	153,842	42,184 (27.4)	28.2	2,891	883 (30.5)	82.8	2.09	Reference
Transgender persons	5,445	1,860 (34.2)	1.2	98	25 (25.5)	2.3	1.34	0.64 (0.43–0.95)***
Persons who inject drugs	37,212	6,425 (17.3)	4.3	254	26 (10.2)	2.4	0.40	0.19 (0.13–0.29)^¶^
Heterosexual males	159,948	44,641 (27.9)	29.9	464	75 (16.2)	7.0	0.17	0.08 (0.06–0.10)^¶^
Heterosexual females	154,598	54,323 (35.1)	36.4	312	57 (18.3)	5.3	0.10	0.05 (0.04–0.07)^¶^
**Total in non–health care facilities**	**703,890**	**202,181 (28.7)**	**100.0**	**4,860**	**1,292 (26.6)**	**100.0**	**0.64**	**—**

**Abbreviations:** aPR = adjusted prevalence ratio; HIV = human immunodeficiency virus.

* Youths are defined as teens and young adults aged 13–24 years.

^†^ Valid HIV tests were defined as tests for which a test result (i.e., positive or negative) was known. Analyses excluded discordant and indeterminate results.

^§^ Included are persons who tested HIV-positive and did not report a previous positive test result, calculated using HIV surveillance verification (if available) or a person’s self-reported previous HIV status.

^¶^ Significant at p<0.001.

** Missing/invalid data were excluded. In the category “HIV tests among youths,” 6,088 (0.73%) records were excluded from gender, 51,424 (6.13%) from race/ethnicity, and 3,073 (0.37%) from test setting. In the category “newly diagnosed HIV infection among youths,” four (0.13%) were excluded from gender, 62 (2.09%) from race/ethnicity, and 16 (0.54%) from test setting.

**^††^** Includes Asian, American Indian or Alaska Native, and Native Hawaiian or Pacific Islander.

^§§^ Significant at p<0.01.

**^¶¶^** Target population data are available only for tests performed in non–health care facilities and not for tests performed in health care facilities. Therefore, these data come from non–health care facilities only. Records that specified other target population and those with missing/invalid data were excluded. In the category “HIV tests among youths,” 33,703 (16.67%) records that specified other target population and 19,045 (9.42%) records with missing/invalid data were excluded. In the category “newly diagnosed HIV infection among youths,” 208 (16.10%) records that specified other target population and 18 (1.39%) records with missing/invalid data were excluded.

*** Significant at p<0.05.

Among 2,973 youths who received a new diagnosis of HIV infection, 1,955 (66%) were linked to HIV medical care within 90 days of diagnosis, and 1,871 (63%) were interviewed for partner services (Table 2). Compared with the South (64%), the prevalence of being linked to HIV medical care within 90 days of diagnosis was lower in the Midwest (49%; aPR = 0.81) but higher in the Northeast (78%; aPR = 1.18), West (77%; aPR = 1.16), and U.S. dependent areas*** (86%; aPR = 1.50) (Table 2). The prevalence of being interviewed for partner services in the South was 62%; compared with the South, this prevalence was higher in the West (70%; aPR = 1.09) and the U.S. dependent areas (84%; aPR = 1.34) (Table 2).

**TABLE 2:** Linkage to HIV medical care and interviews for partner services among newly diagnosed HIV-positive youths,* by selected characteristics — United States, Puerto Rico, and the U.S. Virgin Islands, 2015

Characteristic	No. newly diagnosed HIV infections,^†^ (% of total)	Linked to HIV medical care within 90 days of diagnosis^§^			Interviewed for partner services^¶^		
		No. (% of subgroup)	aPR (95% CI)	No. missing linkage info. (%)	No (%)	aPR (95% CI)	No. missing linkage info. (%)
**Age group (yrs)**							
13–19	415 (14.0)	263 (63.4)	0.95 (0.88–1.03)	121 (29.2)	258 (62.2)	0.98 (0.90–1.06)	88 (21.2)
20–24	2,558 (86.0)	1,692 (66.1)	Reference	582 (22.8)	1,613 (63.1)	Reference	475 (18.6)
Gender**							
Male	2,661 (86.9)	1,758 (66.1)	Reference	624 (23.5)	1,693 (63.6)	Reference	500 (18.8)
Female	256 (8.6)	163 (63.7)	1.00 (0.91–1.10)	68 (26.6)	147 (57.4)	0.92 (0.83–1.03)	47 (18.4)
Transgender	52 (1.8)	31 (59.6)	0.89 (0.72–1.10)	11 (21.2)	28 (53.9)	0.86 (0.67–1.11)	16 (30.8)
Race/Ethnicity**							
White	440 (15.1)	302 (68.6)	1.08 (0.99–1.16)	93 (21.1)	275 (62.5)	0.97 (0.88–1.05)	74 (16.8)
Black or African American	1,727 (59.3)	1,085 (62.8)	Reference	437 (25.3)	1,074 (62.2)	Reference	355 (20.6)
Hispanic or Latino	626 (21.5)	460 (73.5)	1.04 (0.97–1.10)	127 (20.3)	424 (67.7)	1.00 (0.94–1.08)	89 (14.2)
Other**^††^**	67 (2.3)	52 (77.6)	1.11 (0.98–1.26)	9 (13.4)	40 (59.7)	0.89 (0.73–1.08)	13 (19.4)
Multiple races	51 (1.8)	32 (62.7)	0.96 (0.78–1.18)	15 (29.4)	29 (56.9)	0.88 (0.70–1.11)	12 (23.5)
Test setting**							
Health care facility	1,665 (56.3)	1,093 (65.6)	1.00 (0.95–1.05)	396 (23.8)	1,052 (63.2)	1.01 (0.95–1.07)	302 (18.1)
Non–health care facility	1,292 (43.7)	853 (66.0)	Reference	304 (23.5)	807 (62.5)	Reference	260 (20.1)
**U.S. Census region**							
Northeast	373 (12.5)	292 (78.3)	1.18 (1.11–1.27)^§§^	49 (13.1)	245 (65.7)	1.05 (0.97–1.15)	81 (21.7)
Midwest	439 (14.8)	216 (49.2)	0.81 (0.73–0.90)^§§^	157 (35.8)	240 (54.7)	0.85 (0.77–0.94)	68 (15.5)
South	1,687 (56.7)	1,077 (63.8)	Reference	423 (25.1)	1,049 (62.2)	Reference	383 (22.7)
West	431 14.5)	333 (77.3)	1.16 (1.09–1.25)^§§^	68 1(5.8)	301 (69.8)	1.09 (1.01–1.18)^¶¶^	26 (6.0)
U.S. dependent areas	43 (1.4)	37 (86.0)	1.50 (1.29–1.74)^§§^	6 14.0)	36 (83.7)	1.34 1.14–1.58)^§§^	5 (11.6)
**HIV prevalence**							
High	1,923 (64.7)	1,48 (70.1)	Reference	388 20.2)	1,217 (63.3)	Reference	353 (18.4)
Medium	981 (33.0)	545 (55.6)	0.83 (0.78–0.89)^§§^	311 31.7)	590 (60.1)	0.99 0.92–1.06)	208 (21.2)
Medium-low/Low	69 (2.3)	62 (89.9)	1.30 (1.18–1.44)^§§^	4 5.8)	64 (92.8)	1.64 1.49–1.81)^§§^	2 (2.9)
**Overall total**	**2,973**	**1,955 (65.8)**	**—**	**703 23.7)**	**1,871 (62.9)**	**—**	**563 (18.9)**

**Abbreviations:** aPR = adjusted prevalence ratio; HIV = human immunodeficiency virus.

* Youths are defined as teens and young adults aged 13–24 years.

^†^ Includes persons who tested HIV-positive during the current test and were not found to be previously reported in the health department jurisdiction’s HIV surveillance system or self-reported not having a previous HIV-positive test result if surveillance system verification was not available.

^§^ Linkage to HIV medical care within 90 days of diagnosis means confirmation that the person attended their first HIV medical care appointment within 90 days of their diagnosis.

^¶^ “Partner services” is a process through which HIV-infected persons are interviewed to elicit information about their partners, who can then be confidentially notified of their possible exposure or potential risk and offered services that can protect the health of partners and prevent HIV transmission to others.

** Missing or invalid data were excluded. For the category “linked to HIV medical care,” three records were excluded from gender, 24 from race/ethnicity, and nine from test setting. For the category “interviewed for partner services,” three records were excluded from gender, 29 from race/ethnicity, and 12 from test setting.

**^††^** Includes Asian, American Indian or Alaska Native, and Native Hawaiian or Pacific Islander.

^§§^ Significant at p<0.001.

^¶¶^ Significant at p<0.05.

Among 4,884 HIV infections identified among youths, 1,911 (39%) had been previously diagnosed, and 1,749 (92%) of these youths with previously diagnosed infection were not in HIV medical care at the time of testing (Table 1) (Table 3). Among the 1,749 youths with previously diagnosed infection who were not in HIV medical care, 66% were linked to care within 90 days of diagnosis. Compared with the South, where the prevalence of being linked to care was 61%, the prevalence was higher in the Northeast (77%; aPR = 1.30), Midwest (74%; aPR = 1.21), and the West (83%; aPR = 1.33).

**TABLE 3:** Linkage to HIV medical care among previously diagnosed HIV-positive youths,* by selected characteristics, United States, Puerto Rico, and the U.S. Virgin Islands, 2015

Characteristic	No. previously diagnosed HIV infections^†^	No. not in HIV medical care at time of current HIV test (%)	Previously diagnosed HIV-positive youths not in HIV medical care at time of this test who were then linked to care^§^		
			No. (%)	aPR (95% CI)	No. missing linkage info (%)
**Age group (yrs)**					
13–19	216	197 (91.2)	134 (68.0)	1.02 (0.93–1.13)	33 (16.8)
20–24	1,695	1,552 (91.6)	1,023 (65.9)	Reference	350 (22.6)
**Gender** ^¶^					
Male	1,615	1,474 (91.3)	986 (66.9)	Reference	311 (21.1)
Female	256	239 (93.4)	149 (62.3)	0.94 (0.85–1.05)	62 (25.9)
Transgender	31	27 (87.1)	19 (70.3)	1.02 (0.80–1.30)	7 (25.9)
**Race/Ethnicity^¶^**					
White	221	213 (96.4)	156 (73.2)	1.09 (0.99–1.20)	40 (18.8)
Black or African American	1,328	1,192 (89.8)	775 (65.0)	Reference	268 (22.5)
Hispanic or Latino	243	232 (95.5)	167 (72.0)	1.06 (0.96–1.17)	43 (18.5)
Other**	26	26 (100.0)	20 (76.9)	1.07 (0.85–1.33)	3 (11.5)
Multiple races	15	14 (93.3)	7 (50.0)	0.72 (0.43–1.20)	5 (35.7)
**Test setting^¶^**					
Health care facility	1,433	1,304 (91.0)	868 (66.6)	1.01 (0.93–1.10)	280 (21.5)
Non-health care facility	470	438 (93.2)	287 (65.5)	Reference	100 (22.8)
**U.S. Census region**					
Northeast	129	112 (86.8)	86 (76.8)	1.30 (1.16–1.46)^††^	19 (17.0)
Midwest	325	276 (84.9)	203 (73.6)	1.21 (1.11–1.33)^††^	42 (15.2)
South	1,294	1,210 (93.5)	742 (61.3)	Reference	306 (25.3)
West	156	144 (92.3)	120 (83.3)	1.33 (1.20–1.47)^††^	16 (11.1)
U.S. dependent areas	7	7 (100.0)	6 (85.7)	1.29 (0.93–1.78)	0 (0.0)
**HIV prevalence**					
High	1043	942 (90.3)	629 (66.8)	Reference	188 (20.0)
Medium	854	797 (93.3)	521 (65.4)	1.07 (1.00–1.15)	193 (24.2)
Medium-low/Low	14	10 (71.4)	7 (70.0)	0.95 (0.66–1.38)	2 (20.0)
**Overall total**	**1,911**	**1,749 (91.5)**	**1,157 (66.2)**	**—**	**383 (21.9)**

**Abbreviations:** aPR = adjusted prevalence ratio; HIV = human immunodeficiency virus.

* Youths are defined as teens and young adults aged 13−24 years.

^†^ Previously diagnosed HIV infections include those who tested HIV-positive during the current test and were found to be previously reported in the health department’s HIV surveillance system or self-reported having a previous HIV-positive test result if the surveillance system verification is not available.

^§^ Linkage to HIV medical care within 90 days of diagnosis means confirmation that the person attended their first HIV medical care appointment within 90 days of receiving their diagnosis.

^¶^ Missing or invalid data were excluded. In the category “previously diagnosed HIV infections,” nine records were excluded from gender, 78 from race/ethnicity, and eight from test setting. In the category “linked to care,” three records were excluded from gender, 32 from race/ethnicity, and two from test setting.

** Includes Asian, American Indian or Alaska Native, and Native Hawaiian or Pacific Islander.

^††^ Significant at p<0.001.

## Discussion

Among youths living with HIV infection, testing and partner services are important strategies for diagnosis and prompt linkage to medical care so they can achieve viral suppression and reduce their risk for transmission to others. A national surveillance study determined that 92% of new infections in 2009 were acquired from persons with HIV who were not in medical care, underscoring the importance of early diagnosis and ongoing care and treatment (*6*). Given that approximately half of young MSM living with HIV are unaware of their status, and that the testing rates among youths are relatively low, improving the number of youths who are tested is of high importance (*3*,*4*). Including HIV testing as part of routine medical care for youths is key to increasing early diagnosis, and a health care provider’s testing recommendation is the most important predictor of testing among adolescents at risk for HIV infection (*7*). It is especially important to test young MSM because they are most disproportionately affected by HIV. In this analysis, young MSM, who accounted for 83% of new HIV infection diagnoses among all youths in non–health care facilities, received 28% of the tests. Expanding testing in places where youths might interact with the health care system, and providing youth- and lesbian/gay/bisexual/transgender-friendly services, might increase testing among youths, especially young MSM (*8*).

By 2020, national goals call for linking 85% of persons who received a new diagnosis of HIV infection to medical care within 30 days of diagnosis (*1*). Findings from this report indicate that approximately two thirds (66%) of youths who received a diagnosis of HIV infection in 2015 through CDC-funded HIV testing programs were linked to care within 90 days of diagnosis. Among youths who received a new HIV infection diagnosis, 63% were interviewed for partner services. The greatest need for improvement appears to be in the South and the Midwest, where rates of linkage and interviews for partner services were relatively low. These low rates are particularly concerning for the South because in 2015, approximately 52% of new diagnoses of HIV infection in the United States occurred in the region (*9*).

In this analysis, 39% of youths who received an HIV-positive test result had already received a diagnosis of HIV infection in the past, and 92% of those persons were not in HIV medical care at the time of the test. Youths who have previously received a diagnosis of HIV infection and are willing to receive a test present an important HIV treatment opportunity. Youths might face multiple barriers to treatment, such as consent and confidentiality, inability to pay for services, housing instability, stigma, lack of transportation, and mental health and substance use, which might need to be addressed for successful linkage and retention to care (*8*).

The findings in this report are subject to at least three limitations. First, findings describe CDC-funded HIV tests only and are not generalizable to all youths in the United States. Second, linkage data include records with missing or invalid data in the denominator, and therefore probably underestimate the percentage of persons linked to care. Finally, when surveillance data are unavailable to verify prior HIV status, the number of new positive results might be overestimated if clients inaccurately report a previous negative HIV status.

Increasing the number of HIV tests among youths at risk for HIV and increasing regular retesting among these youths is essential for reducing HIV infection in this vulnerable population. This could be accomplished through a combined strategy of routine HIV testing among youths, especially young men, in health care settings, and targeted testing in places where youths at risk for HIV infection congregate. CDC currently funds 120 CBOs to provide targeted testing to the populations most at risk for HIV infection; 30 of these CBOs specifically serve young MSM and transgender persons of color (*10*). Additional measures are needed to encourage health care providers to include HIV testing as a routine part of health care for youths. Schools can also play an important role in facilitating access to HIV testing for school-aged youth. Toward this end, CDC works with 18 state education agencies and 17 local education agencies to connect youths to community-based services, including HIV testing. Increased measures are also needed to ensure that youths who receive positive test results for HIV are rapidly linked to and retained in appropriate medical care, including early initiation of antiretroviral therapy.

**Summary**

**What is already known about this topic?**

In 2014, 70% of teens and young adults aged 13–24 (youths) who received a diagnosis of HIV infection were young men who have sex with men (MSM), 52% of whom were unaware of their infection. HIV testing rates are low among youths, with 22% of sexually active high school students and 33% of young adults aged 18–24 years reporting ever having received an HIV test.

**What is added by this report?**

Analysis of 2015 data on CDC-funded HIV tests and HIV prevention services from 61 health departments and 123 community-based organizations indicated that young MSM, who accounted for 83% of new diagnoses of HIV infection among all youths in non–health care facilities, received 28% of the tests in these settings. The 2020 national goal is to link at least 85% of HIV-positive persons to HIV medical care within 30 days of diagnosis; in this analysis, 66% of youths whose test results were positive for HIV were linked to care within 90 days of diagnosis. HIV disproportionately affects youths in the South, where linkage rates were among the lowest in the United States.

**What are the implications for public health practice?**

Increasing HIV testing among youths at risk for HIV infection is essential for reducing infections in this vulnerable population. This can be accomplished through a combined strategy of routine HIV testing of youths, especially young men, in health care settings and targeted testing in places where youths at risk for HIV infection congregate. Schools can also play an important role in facilitating access to HIV testing for school-aged youths. Increased measures are needed to ensure that youths testing positive for HIV are rapidly linked to and retained in appropriate medical care, including early initiation of antiretroviral therapy.

## Footnotes

* Non–health care facilities are settings where HIV testing is performed using a targeted testing strategy rather than a routine screening strategy. Examples of non–health care facilities include HIV testing sites and community settings.

† CDC-funded partners include health departments in 50 states, the District of Columbia, Puerto Rico, the U.S. Virgin Islands, and eight directly funded city/county health departments (Baltimore, Maryland; Chicago, Illinois; Fulton County, Georgia; Houston, Texas; Los Angeles County, California; New York City, New York; Philadelphia, Pennsylvania; and San Francisco, California) and 123 directly funded community-based organizations. Community-based organizations report their National HIV Prevention Program Monitoring and Evaluation HIV testing data to their jurisdiction’s health department who then submit them to CDC.

§ An HIV test is defined as the performance of one or more HIV tests to determine a person’s HIV infection status. A person might be tested once (e.g., one rapid test or one conventional test) or multiple times (e.g., one rapid test followed by one conventional test to confirm a preliminary HIV-positive test result).

¶ Linkage to HIV medical care within 90 days of diagnosis means confirmation that the person attended their first HIV medical care appointment within 90 days of their HIV test date.

** “Partner services” is a process through which HIV infected persons are interviewed to elicit information about their partners, who can then be confidentially notified of their possible exposure or potential risk and offered services that can protect the health of partners and prevent HIV transmission to others.

†† Jurisdictions are grouped by HIV prevalence as determined by the number of persons living with diagnosed HIV infection in 2013: high: ≥20,000; medium: 4,000–19,999; medium-low/low: ≤3,999. High prevalence jurisdictions include the following: California, Los Angeles, San Francisco; Florida; Georgia, Fulton County (Atlanta); Chicago, Illinois; Maryland, Baltimore; New Jersey; New York, New York City; North Carolina; Pennsylvania, Philadelphia; Texas, Houston; and Virginia. Medium prevalence jurisdictions include Alabama; Arizona; Arkansas; Colorado; Connecticut; District of Columbia; Indiana; Kentucky; Louisiana; Massachusetts; Michigan; Minnesota; Mississippi; Missouri; Nevada; Ohio; Oklahoma; Oregon; Puerto Rico; South Carolina; Tennessee; Washington; and Wisconsin. Medium-low/low prevalence jurisdictions include Alaska; Delaware; Hawaii; Idaho; Iowa; Kansas; Maine; Montana; Nebraska; New Hampshire; New Mexico; Rhode Island; South Dakota; U.S. Virgin Islands; Utah; Vermont; West Virginia; and Wyoming.

§§ Target population data are available only for tests performed in non–health care facilities and not for tests performed in health care facilities.

¶¶ Other races/ethnicities include Asian, American Indian or Alaska Native, and Native Hawaiian or Pacific Islander.

*** For this report, U.S. dependent areas include only Puerto Rico and the U.S. Virgin Islands.

## References

[1] Office of National AIDS Policy. National HIV/AIDS strategy for the United States: updated to 2020. Washington, DC: Office of National AIDS Policy; 2015. https://obamawhitehouse.archives.gov/sites/default/files/docs/national_hiv_aids_strategy_update_2020.pdf

[2] CDC. Diagnoses of HIV infection among adolescents and young adults in the United States and 6 dependent areas, 2010–2014. Atlanta, GA: US Department of Health and Human Services, CDC; 2016. https://www.cdc.gov/hiv/pdf/library/reports/surveillance/cdc-hiv-surveillance-supplemental-report-vol-21-3.pdf

[3] Singh S, Song R, Satcher Johnson A, McCray E, Hall I. HIV incidence, prevalence, and undiagnosed infections in men who have sex with men. Presented at the Conference on Retroviruses and Opportunistic Infections, Seattle, Washington; February 13–16, 2017. http://www.hivdent.org/_CROI2017/HIV%20Incidence.pdf

[4] Van Handel M, Kann L, Olsen EO, Dietz P. HIV testing among US high school students and young adults. Pediatrics 2016;137:e20152700. 10.1542/peds.2015-2700 26787047

[5] Branson BM, Handsfield HH, Lampe MA, Revised recommendations for HIV testing of adults, adolescents, and pregnant women in health-care settings. MMWR Recomm Rep 2006;55(No. RR-14). 16988643

[6] Skarbinski J, Rosenberg E, Paz-Bailey G, Human immunodeficiency virus transmission at each step of the care continuum in the United States. JAMA Intern Med 2015;175:588–96. 10.1001/jamainternmed.2014.8180 25706928

[7] Murphy DA, Mitchell R, Vermund SH, Futterman D; HIV/AIDS Research Network. Factors associated with HIV testing among HIV-positive and HIV-negative high-risk adolescents: the REACH Study. Pediatrics 2002;110:e36. 10.1542/peds.110.3.e36 12205286

[8] Zanoni BC, Mayer KH. The adolescent and young adult HIV cascade of care in the United States: exaggerated health disparities. AIDS Patient Care STDS 2014;28:128–35. 10.1089/apc.2013.0345 24601734 PMC3948479

[9] CDC. HIV surveillance report: diagnoses of HIV infection in the United States and dependent areas, 2015. Atlanta, GA: US Department of Health and Human Services, CDC; 2016. https://www.cdc.gov/hiv/pdf/library/reports/surveillance/cdc-hiv-surveillance-report-2015-vol-27.pdf

[10] CDC. Funding announcement PS17–1704: comprehensive high-impact HIV prevention projects for young men of color who have sex with men and young transgender persons of color. Atlanta, GA: US Department of Health and Human Services, CDC; 2016. https://www.cdc.gov/hiv/funding/announcements/ps17-1704/index.html

